# BMC Gastroenterology reviewer acknowledgement 2014

**DOI:** 10.1186/s12876-015-0238-x

**Published:** 2015-02-05

**Authors:** Magdalena Morawska

**Affiliations:** BioMed Central, Floor 6, 236 Gray′s Inn Road, London, WC1X 8HB UK

## Abstract

The editors of BMC Gastroenterology would like to thank all our reviewers who have contributed to the journal in Volume 14 (2014).

**Sumadiono**

Indonesia

**Monica Acalovschi**

Romania

**Stefan Acosta**

Sweden

**Yuichi Adachi**

Japan

**Luigi Adinolfi**

Italy

**Silvia Affo**

USA

**Nishat Afroz**

India

**Vikas Agarwal**

India

**Rakesh Aggarwal**

India

**Ashish Aggarwal**

USA

**Alessio Aghemo**

Italy

**Nikhil Agrawal**

India

**Ferdinando Agresta**

Italy

**Siv Ahrne**

Sweden

**Guruprasad Aithal**

UK

**Holly Algood**

USA

**Alina Allen**

USA

**Piero Luigi Almasio**

Italy

**Amin Alousi**

USA

**Akif Altinbas**

Turkey

**Deepak Amarapurkar**

India

**Samad Amini-Bavil-Olyaee**

USA

**Javier Ampuero**

Spain

**Ashwin Ananthakrishnan**

USA

**Emma Anderson**

UK

**Pietro Andreone**

Italy

**Francesco Angelico**

Italy

**Paul Angulo**

USA

**Donald Anthony**

USA

**Kunihiko Aoyagi**

Japan

**Teoman Apan**

Turkey

**Tomio Arai**

Japan

**Joop Arends**

Netherlands

**Juan Armendariz-Borunda**

Mexico

**Alessandro Armuzzi**

Italy

**Marcella Arru**

Italy

**David Assis**

USA

**Ajax Atta**

Brazil

**Phil Austin**

UK

**Ali Azarm**

USA

**Lukas Büngens**

Germany

**Yi Ba**

China

**Hideo Baba**

Japan

**Zahra Babaei**

Iran

**Alfa Hsing Chen Bai**

Hong Kong

**Sarah Ballou**

USA

**Ji Young Bang**

USA

**Bulent Baran**

Turkey

**Varenka J Barbero Becerra**

Mexico

**Marisa Bare**

Spain

**Savio Barreto**

India

**Anushka Baruah**

USA

**Guido Basilisco**

Jamaica

**Daniela Basso**

Italy

**Joana Bastos**

Portugal

**Tom Bates**

UK

**Ian Beales**

UK

**Andrew Beggs**

UK

**Stefano Bellentani**

Italy

**Massimo Bellini**

Italy

**Amine Benkabbou**

Morocco

**Tim Benning**

Netherlands

**Shay Ben-Shachar**

Israel

**Charles Bernstein**

Canada

**Simona Bertoni**

Italy

**Mirko Bertozzi**

Italy

**Annalisa Berzigotti**

Spain

**Emilie Bessède**

France

**Jan Best**

Germany

**Mark Bettington**

Australia

**Aneel Bhangu**

UK

**Bhushan Bhole**

India

**Federico Biagi**

Italy

**Veerle Bieghs**

Germany

**Alberto Biondi**

Italy

**Edward Bixler**

USA

**Wojciech Blonski**

USA

**Anneloes Bohte**

Netherlands

**Bernd Bokemeyer**

Germany

**Eduardo Bondan**

Brazil

**Ole Bonderup**

Denmark

**Joel Bornstein**

Australia

**Linda Jw Bosch**

Netherlands

**Xavier Bossuyt**

Belgium

**Alex Bottle**

UK

**Christopher Bowlus**

USA

**Anders Boyd**

France

**Patrizia Brigidi**

Italy

**Giuseppe Brisinda**

Italy

**Aurore Brondex**

France

**Jan Bruensing**

Germany

**Sylvia Brugman**

Netherlands

**Stanislas Bruley Des Varannes**

France

**Christine Budke**

USA

**Anthony Buisson**

France

**Christophe Burucoa**

France

**Peter Bytzer**

Denmark

**Karel Caca**

Germany

**Sanjun Cai**

China

**Roberto Calcedo**

USA

**Vincenza Calvaruso**

Italy

**M. Constanza Camargo**

USA

**Ali Canbay**

Germany

**Rocco Cappellesso**

Italy

**Maria Cappello**

Italy

**Gabriele Capurso**

Italy

**Paolo Caraceni**

Italy

**Andrea Cariati**

Italy

**Bertrand Cariou**

France

**Ignazio Castagliuolo**

Italy

**Renato Caviglia**

Italy

**Sang Hoon Cha**

South Korea

**Heyson Chan**

Hong Kong

**Walter Chan**

USA

**David Chang**

UK

**Christophe Chassard**

Switzerland

**Hans Chen**

China

**Yung-Tai Chen**

Taiwan

**Alfred Cheng**

Hong Kong

**Daye Cheng**

China

**Toshimi Chiba**

Japan

**Yi-Chun Chiu**

Taiwan

**Minsig Choi**

USA

**Jinah Choi**

USA

**Bruno Christ**

Germany

**Chen Shuan Chung**

Taiwan

**Marco Cicardi**

Italy

**Rachele Ciccocioppo**

Italy

**Francois X Claret**

USA

**Irene Coati**

Italy

**Jeremy Cobbold**

UK

**Lori Coburn**

USA

**Audrey Coilly**

France

**Daniel Compagno**

Argentina

**Jason Coombes**

UK

**Gareth Corbett**

UK

**Renato Costi**

Italy

**José Cotter**

Portugal

**Jason Craggs**

USA

**Timothy Craig**

USA

**Cesare Cremon**

Italy

**Antonino Crinò**

Italy

**Piotr Czauderna**

Poland

**Chia-Yen Dai**

Taiwan

**Heather Dawson**

Switzerland

**Nicola De Bortoli**

Italy

**Arjuna De Silva**

Sri Lanka

**Antonio De Vincentis**

Italy

**Thomas De Wijkerslooth**

Netherlands

**Chris Dekaney**

USA

**Ihsan Ekin Demir**

Germany

**Mark Deneau**

Canada

**Mohammad Derakhshan**

UK

**Wim Derave**

Belgium

**Renumathy Dhanasekaran**

USA

**Puneet Dhar**

India

**Francesco Di Mario**

Italy

**Massimiliano Di Pietro**

UK

**Antonio Di Sabatino**

Italy

**Christoph Dietrich**

Germany

**John Dillon**

UK

**Linda Dirven**

Netherlands

**Sanjay Dixit**

UK

**Massimo Dominici**

Italy

**Henry Dong**

USA

**Jia-Hong Dong**

China

**Paola Dongiovanni**

Italy

**Steven Dooley**

Germany

**Maria Pina Dore**

Italy

**Richard Douard**

France

**Alexander Dowli**

USA

**Peter Draganov**

USA

**Chelsea Drennan**

USA

**Caigan Du**

Canada

**Pradeep Dudeja**

USA

**Dan Dumitrascu**

Romania

**Ximena Duque**

Mexico

**Dayna Early**

USA

**Mattias Ekstedt**

Sweden

**Hicham El Alaoui**

France

**Karima El Rhazi**

Morocco

**Abdul Elkadri**

Canada

**Magdy El-Salhy**

Norway

**Anne Line Engsbro**

Denmark

**Munechika Enjoji**

Japan

**Bang Wool Eom**

South Korea

**Rune Erichsen**

Denmark

**Mert Erkan**

Germany

**M Estep**

USA

**Antongiulio Faggiano**

Italy

**Ezio Falletto**

Italy

**Klaudia Farkas**

Hungary

**Giammarco Fava**

Italy

**Francesco Feo**

Italy

**Juan Manuel Fernández**

Spain

**Conrado M Fernandez Rodriguez**

Spain

**Jose Fernandez-Cebrian**

Spain

**Davide Festi**

Italy

**Carmine Finelli**

Italy

**Mitchell Fink**

USA

**Gionata Fiorino**

Italy

**Marco P. Fisichella**

USA

**Ruggiero Francavilla**

Italy

**Francesco Franceschi**

Italy

**Luca Frulloni**

Italy

**Ezequiel Fuentes-Panana**

Mexico

**Takeshi Fujita**

Japan

**Manuele Furnari**

Italy

**Anthony Gamboa**

USA

**Carmelo Garcia-Monzon**

Spain

**Manuela Gariboldi**

Italy

**Alfredo Garofalo**

Italy

**Antonio Gasbarrini**

Italy

**Christoph Gasche**

Austria

**Jeremie Gautheron**

Germany

**Maria Gazouli**

Greece

**Karel Geboes**

Belgium

**Krisztina Gecse**

Hungary

**Lorenzo Gemignani**

Italy

**Sotirios Georgopoulos**

Greece

**Uday Ghoshal**

India

**Peter Gillett**

UK

**Saulius Girnius**

USA

**Gabriel Glockzin**

Germany

**Petra Anna Golovics**

Hungary

**Ana M Gomez Pedrero**

Spain

**Fernando Gomollón García**

Spain

**Yihong Gong**

China

**Stevan Gonzalez**

USA

**Jeroen Goos**

Netherlands

**Vinod Gopalan**

Australia

**Ilyssa Gordon**

USA

**Eriko Goto**

Japan

**Corinne Gower-Rousseau**

France

**Richa Goyal**

India

**Sergio Gradilone**

USA

**Peter Green**

USA

**Christian Grieser**

Germany

**Stefania Grimaudo**

Italy

**Axel Grothey**

USA

**Frank Grünhage**

Germany

**Stefano Guandalini**

USA

**Shruti Guhasarkar**

India

**Arjun Gupta**

USA

**Prabodh Gupta**

USA

**Sebastian Haag**

Germany

**Yoshio Haga**

Japan

**Lutz Hamann**

Germany

**Amira Hamzaoui**

Tunisia

**Jimin Han**

South Korea

**Yuyan Han**

USA

**Kenichi Harada**

Japan

**Simon Hart**

UK

**Muhammad Hasan**

USA

**Ammar Hassan**

USA

**John Hayman**

Australia

**Sugata Hazra**

USA

**Tianlin He**

China

**Paul Henderson**

UK

**Fernando Herbella**

Brazil

**Ulf Herbers**

Germany

**Rolando Hernández-Muñoz**

Mexico

**Klaus Herrlinger**

Germany

**Kerstin Herzer**

Germany

**Ludwig Heuss**

Switzerland

**Susumu Hijioka**

Japan

**Ida Hilmi**

Malaysia

**Kaveh Hoda**

USA

**Iva Hojak**

Croatia

**James Hollis**

USA

**Masaki Honda**

Japan

**Sam Hong**

USA

**Charlotte Hor Henrichsen**

Spain

**Kazutoshi Hori**

Japan

**Akira Horiuchi**

Japan

**Jan-Sing Hsieh**

Taiwan

**Jian Hu**

Hong Kong

**Chun-Chieh Huang**

Taiwan

**Wolfgang Huber**

Germany

**Patrick Hughes**

Australia

**Ivan Fn Hung**

Hong Kong

**Niall Patrick Hyland**

Ireland

**Woo Jin Hyung**

South Korea

**Enzo Ierardi**

Italy

**Joep Ijspeert**

Netherlands

**Akira Imoto**

Japan

**Osamu Inatomi**

Japan

**Giuseppe Ingravallo**

Italy

**Pasquale Innominato**

France

**Paola Iovino**

Italy

**Hirotoshi Ishiwatari**

Japan

**Norio Isoda**

Japan

**Robert Issenman**

Canada

**Takao Itoi**

Japan

**Hrvoje Ivekovic**

Croatia

**Hartmut Jaeschke**

USA

**Anahita Jalilvand**

USA

**Lina Jansen**

Germany

**Thierry Jarde**

Australia

**Johannes Jeekel**

Netherlands Antilles

**Christian**

Jenssen Germany

**Hui Jiang**

UK

**Acer Jiang**

China

**Sabita Jiwnani**

India

**Lloris Jm**

Spain

**Nicolas Jonckheere**

France

**Michael Jones**

Australia

**Bharat Joshi**

USA

**Hwoon-Yong Jung**

South Korea

**Ali Kabir**

Iran

**Tatehiro Kagawa**

Japan

**Alisan Kahraman**

Germany

**Marko Kalliomäki**

Finland

**Sunjeev Kamboj**

UK

**Terumi Kamisawa**

Japan

**Nobuo Kanai**

Japan

**Tatsuo Kanda**

Japan

**Umadevi Kandasamy**

China

**Hyoun Woo Kang**

South Korea

**Lakshmi Kannan**

USA

**Konstantinos Kantartzis**

Germany

**Sung-Shuo Kao**

Taiwan

**Paulo Kassab**

Brazil

**Shingo Kato**

Japan

**Konstantinos Katsanos**

Greece

**Seymour Katz**

USA

**Gurjeet Kaur**

Malaysia

**Pawel Kawalec**

Poland

**Laurie Keefer**

USA

**Simon Keely**

Australia

**Daniel Keszthelyi**

Netherlands

**Shara Ket**

UK

**Martin Keuchel**

Germany

**Muhammad Khurram**

Pakistan

**Gwang Ha Kim**

South Korea

**Jae Kim**

South Korea

**Seung Up Kim**

South Korea

**Baek-Hui Kim**

South Korea

**Lajos Kiss**

Hungary

**Masayuki Kitano**

Japan

**Janis Klovins**

Latvia

**Johannes Kluwe**

Germany

**Mate Knabe**

Germany

**C M Frank Kneepkens**

Netherlands

**Hideki Kobara**

Japan

**Taku Kobayashi**

Japan

**Oliver Owen Koch**

Austria

**Rakesh Kochhar**

India

**Motohiro Kojima**

Japan

**Kaija-Leena Kolho**

Finland

**Vani Konda**

USA

**Hanne Konradsen**

Denmark

**Christos Kontos**

Greece

**Marcela Kopacova**

Czech Republic

**Uri Kopylov**

Canada

**Uri Kopylov**

Israel

**John Koskinas**

Greece

**Aletta Kraneveld**

Netherlands

**Wolfgang Kratzer**

Germany

**Andi Krumbholz**

Germany

**Anja Kühl**

Germany

**Roland Kuiper**

Netherlands

**Seray Külcü Çakmak**

Turkey

**Hemant Kulkarni**

USA

**Teru Kumagi**

Japan

**Manoj Kumar**

India

**Juozas Kupcinskas**

Lithuania

**Aysegul Kuskucu**

Turkey

**Vincenzo La Mura**

Italy

**Conor Lahiff**

Ireland

**Ming-Wei Lai**

Taiwan

**Terry Lairmore**

USA

**Peter Lakatos**

Hungary

**Brian Lam**

USA

**Kristina Lamas**

Sweden

**Malinee Laopaiboon**

Thailand

**Ryan Law**

USA

**Cedric Le May**

France

**Sang-Jeon Lee**

South Korea

**Yeong Yeh Lee**

Malaysia

**Philippe Lehours**

France

**Michael Leitzmann**

USA

**Marcis Leja**

Latvia

**Cosmas Rinaldi Lesmana**

Indonesia

**William Letson**

USA

**Barrett Levesque**

USA

**Philippe Lévy**

France

**James Lewis**

USA

**Ding-You Li**

USA

**Aiwu Li**

China

**Chuang-Chi Liaw**

Taiwan

**Jingmei Lin**

USA

**Hans Lin**

Taiwan

**Marcelo Linhares**

Brazil

**Chia-Yuan Liu**

Taiwan

**Gin-Ho Lo**

Taiwan

**Amedeo Lonardo**

Italy

**Dayami Lopez**

USA

**Albert Lowenfels**

USA

**M Isabel Lucena**

Spain

**Claudio Luchini**

Italy

**Alexander Lukasz**

Germany

**Tao Luo**

China

**Holger Lutz**

Germany

**Chang-Sheng Ma**

China

**Benjamin Maasoumy**

Germany

**Mariana Machado**

Portugal

**Lloyd Mack**

Canada

**Subha Madhavan**

USA

**Mohammad Madhoun**

USA

**K S Madhusudhan**

India

**Mariantonia Maglio**

Italy

**Sanjiv Mahadeva**

Malaysia

**Govind Makharia**

India

**Atul Malhotra**

Australia

**Shyamapada Mandal**

India

**Hendrik Manner**

Germany

**Spilios Manolakopoulos**

Greece

**Aiping Mao**

USA

**Séverine Margeridon**

France

**Athanasios Marinis**

Greece

**Daniele Marrelli**

Italy

**Javier Martin**

Spain

**Allyson**

Martinez USA

**Luca**

Mastracci Italy

**Daiuske Masuda**

Japan

**Ryota Masuzaki**

USA

**Takahisa Matsuda**

Japan

**Hanno Matthaei**

Germany

**Maria Cristina Mazzilli**

Italy

**Andrew Mckay**

Canada

**Colin Mckay**

UK

**Tobias Meister**

Germany

**Anders Meller Donatsky**

Denmark

**Joshua Melson**

USA

**Nahum Méndez-Sánchez**

Mexico

**Fabrice Menegaux**

France

**Shahin Merat**

Iran

**Manuela Merli**

Italy

**Neil Merrett**

Australia

**Evangelos Messaris**

USA

**Tomoki Michida**

Japan

**Erasmo Miele**

Italy

**Ivana Mikolasevic**

Croatia

**Tetsuya Mine**

Japan

**Pramod Mishra**

India

**Benjamin Misselwitz**

Switzerland

**Keigo Mitsui**

Japan

**Mojgan Mohammadi**

Iran

**Tamás Molnár**

Hungary

**Rosa Monno**

Italy

**Laura Montefusco**

Italy

**Silvia Montenegro**

Brazil

**Jong Ho Moon**

South Korea

**Peter Mooney**

UK

**Joaquim Moraes-Filho**

Brazil

**Tom Moreels**

Belgium

**Leticia Moreira**

Spain

**Luca Morini**

Italy

**Yoshinori Morita**

Japan

**Chigusa Morizane**

Japan

**Mahmoud Mosli**

Canada

**Beat Peter Müller-Stich**

Germany

**Malaisamy Muniyandi**

India

**Atsuhiko Murata**

Japan

**Puja Myles**

UK

**Anna Myléus**

Sweden

**Alessio Naccarati**

Italy

**Takeaki Nagamine**

Japan

**Iris Nagtegaal**

Netherlands

**Shan Naidu**

USA

**Hayato Nakagawa**

Japan

**Shigemi Nakajima**

Japan

**Tadashi Nakamura**

Japan

**Udayakumar Navaneethan**

USA

**Rick Nelson**

UK

**Alessandro Neri**

Italy

**Yulia Nevzorova**

Germany

**Siew Ng**

Hong Kong

**Felix Nickel**

Germany

**Scott Nightingale**

Australia

**Mehrdad Nikfarjam**

Australia

**Akiyoshi Nishio**

Japan

**Mark Noar**

USA

**Camilla Nøjgaard**

Denmark

**Kristina Norman**

Germany

**Alireza Norouzi**

Iran

**Fredrik Norstrom**

Sweden

**Oscar Núñez Martínez**

Spain

**Sylvester Chuks Nwokediuko**

Nigeria

**Katsutoshi Obara**

Japan

**Stefan Oehlers**

USA

**Bodil Ohlsson**

Sweden

**Yuki Ohya**

USA

**Shiro Oka**

Japan

**Abdulfatai Olokoba**

Nigeria

**Ehinmidu Olorunmola Joseph**

Nigeria

**Colm O'Morain**

Ireland

**Nikolaj Worm Orntoft**

Denmark

**Cécile Oury**

Belgium

**Nilufer Ozaydin**

Turkey

**Fabio Pace**

Italy

**Ganesh Pai**

India

**Sujoy Pal**

India

**Grazia Palomba**

Italy

**Anand Pandey**

India

**Mária Papp**

Hungary

**Alfonso Papparella**

Italy

**Barbara Pardini**

Czech Republic

**Se Jin Park**

South Korea

**Kt Park**

USA

**Rosa Maria Pascale**

Italy

**Devis Pascut**

Italy

**Michael Pavlides**

UK

**Anna Pecorelli**

Italy

**George Peppas**

Greece

**Montserrat Perez Encinas**

Spain

**Angeles Perez-Aisa**

Spain

**Manuel Perez-Miranda**

Spain

**Giorgio Perilongo**

Italy

**Jonathan Peterson**

USA

**Salvatore Petta**

Italy

**Raffaele Pezzilli**

Italy

**Marco Picardi**

Italy

**Mathieu Piche**

Canada

**Mercedes Pico**

Argentina

**Oreste Pieramico**

Italy

**Hubert Piessevaux**

Belgium

**Alessio Pini Prato**

Italy

**Alejandro Piscoya**

Peru

**Marco Pizzi**

Italy

**Philippe Poirier**

France

**Stefano Pontone**

Italy

**Brian Poole**

USA

**Mahmoudreza Pourkarim**

Belgium

**Akram Pourshams**

Iran

**C Pramesh**

India

**Rashmi Prasad**

Sweden

**Timothy Price**

Australia

**Lani Prideaux**

Australia

**Carlo Pulitano**

Italy

**Giacomo Puppa**

Italy

**Katrina Purcell**

Australia

**Jorge Quiroga**

Spain

**István Rácz**

Hungary

**Robert Raffaniello**

USA

**Jayadev Raju**

Canada

**H Ramesh**

India

**Hari Ramesh**

India

**S.V. Rana**

India

**Bettina M. Rau**

Germany

**Debolina Ray**

USA

**Kevin Reavis**

USA

**Helen Reeves**

UK

**Petr Ricanek**

Norway

**Claudio Ricci**

Italy

**Cristobal Richart**

Spain

**Mark Riddle**

USA

**Rachael Rigby**

UK

**Luciana Rigoli**

Italy

**Cosmeri Rizzato**

Italy

**Barbera Roberta**

Italy

**Martin Roderfeld**

Germany

**Risto Roine**

Finland

**Anders Rönnblom**

Sweden

**Kathleen Ross**

UK

**Thomas Rossi**

USA

**Christophe Rosty**

Australia

**Constance Ruhl**

USA

**Mariagrazia Rumi**

Italy

**Kimberly Ruscher**

USA

**Andrew Ruszkiewicz**

Australia

**Stephen Ryder**

UK

**Sammy Saab**

USA

**Jayesh Sagar**

UK

**David Sahli**

Sweden

**Yutaka Saito**

Japan

**Anne Salonen**

Finland

**John Saltzman**

USA

**Sundeep Saluja**

India

**Dorit Samocha-Bonet**

Australia

**Andres Sanchez-Yague**

Spain

**Olof Sandström**

Sweden

**Fuat Saner**

Germany

**Abbi Saniabadi**

Japan

**Roberto Santambrogio**

Italy

**Aaron Sasson**

USA

**Edoardo Savarino**

Italy

**Stefano Scaringi**

Italy

**Carmelo Scarpignato**

Italy

**Michael Schumann**

Germany

**Andrada Seicean**

Romania

**Gernot Sellge**

Germany

**Lawrence Serfaty**

France

**Dionyssios Sgouras**

Greece

**Chirag N Shah**

India

**Omar Shah**

India

**Tilak Shah**

USA

**Dea Shahinas**

Canada

**Ala Sharara**

Lebanon

**Anamay Sharma**

USA

**Runhua Shi**

USA

**Yuhong Shi**

USA

**Yu-Lueng Shih**

Taiwan

**Takaya Shimura**

Japan

**Cheol Min Shin**

South Korea

**Yasuhiro Shirakawa**

Japan

**Elham Shirvani Dastgerdi**

Germany

**Ivy Shiue**

UK

**Yehuda Shoenfeld**

Israel

**Shailesh Shrikhande**

India

**Nagini Siddavaram**

India

**Parames Sil**

India

**Michele Simbolo**

Italy

**Satu Simell**

Finland

**Harkirat Singh**

USA

**Preet Paul Singh**

USA

**Rajvinder Singh**

Australia

**Shailender Singh**

USA

**Siddharth Singh**

USA

**Iradj Sobhani**

France

**Ajit Sood**

India

**John Souglakos**

Greece

**Bridget Southwell**

Australia

**Valdenia Souza**

Brazil

**Donat Rudolf Spahn**

Switzerland

**Marcel Spanier**

Netherlands

**Ulrich Spengler**

Germany

**Robin Spiller**

UK

**Ioan Sporea**

Romania

**Shanthi Srinivasan**

USA

**Peter Stanich**

USA

**Robert Steele**

UK

**Claudia Stefanutti**

Italy

**Juergen Stein**

Germany

**Jayson Stoffman**

Canada

**Jesse Stondell**

USA

**Kirsten Stone**

USA

**Antoinette Stroup**

USA

**Mitsushige Sugimoto**

Japan

**Ki Tae Suk**

South Korea

**Marianny Sulbaran**

Brazil

**Junfeng Sun**

China

**Xinchen Sun**

China

**Michael Surette**

Canada

**Seth Sweetser**

USA

**Lonnie Swift**

UK

**Jeffrey Swigris**

USA

**Ari Syam**

Indonesia

**James Tabibian**

USA

**Frank Tacke**

Germany

**Wei-Chen Tai**

Taiwan

**Diana Tait**

UK

**Toru Takahashi**

Japan

**Nagio Takigawa**

Japan

**Rupjyoti Talukdar**

India

**Gianluca Tamagno**

Ireland

**Naoto Tamai**

Japan

**Kevin Shyong-Wei Tan**

Singapore

**Manu Tandan**

India

**Raymond Tang**

Hong Kong

**Whitney Tang**

Hong Kong

**Marcel Tantau**

Romania

**Sudeep Tanwar**

UK

**Luc Tappy**

Switzerland

**Deniz Tastemir Korkmaz**

Turkey

**Ryosuke Tateishi**

Japan

**Felix Tellez-Avila**

Mexico

**Richard Ten Broek**

Netherlands

**Christopher Teshima**

Canada

**Jason Theis**

USA

**Gregory Thomas**

UK

**Chengju Tian**

USA

**Kim Timmermans**

Netherlands

**Dina Tiniakos**

Greece

**Jens Tischendorf**

Germany

**Liviu Titu**

UK

**Ervin Toth**

Sweden

**Takashi Toyonaga**

Japan

**Jonel Trebicka**

Germany

**William J. Trickler**

USA

**Andrea Tringali**

Italy

**Emmanuel Tsochatzis**

UK

**Emmanuel Tsochatzis**

Greece

**Toshio Tsuyuguchi**

Japan

**Tao-Hsin Tung**

Taiwan

**Nigel Turner**

Australia

**Antonio Tursi**

Italy

**Koji Uchino**

Japan

**Noriya Uedo**

Japan

**Hirofumi Uto**

Japan

**Francesco Valitutti**

Italy

**Mark Van Baal**

Netherlands

**David Van Der Poorten**

Australia

**Sascha Van Doorn**

Netherlands

**Tom Van Wezel**

Netherlands

**Sina Vatandoust**

Australia

**Maria Velasco**

Spain

**Sander Veldhuyzen Van Zanten**

Canada

**Julien Vergniol**

France

**Piero Vernia**

Italy

**Umberto Vespasiani-Gentilucci**

Italy

**Michael Vieth**

Germany

**Mauro Vigan&Ograve**

Italy

**Cristiane Villela-Nogueira**

Brazil

**Julio Villena**

Argentina

**Manlio Vinciguerra**

UK

**Aron Vincze**

Hungary

**Jiannis Vlachogiannakos**

Greece

**Umberto Volta**

Italy

**Atte Von Wright**

Finland

**Ulrikke Voss**

Sweden

**Amanda Waddell**

USA

**Yasir Waheed**

Pakistan

**Homan Wai**

USA

**Daniel Wall**

UK

**David Wang**

USA

**Fusheng Wang**

China

**Jin-Town Wang**

Taiwan

**Hua Wang**

China

**Mamoru Watanabe**

Japan

**Ketut Dewi Kumara Wati**

Indonesia

**Rabindra Watson**

USA

**Jacques Weill**

France

**Martin-Walter Welker**

Germany

**John Windsor**

New Zealand

**Tibor Wittmann**

Hungary

**Sunny Hei Wong**

Hong Kong

**Vincent Wong**

Hong Kong

**Jeremy Woodward**

UK

**Marcus-Alexander Wörns**

Germany

**Alexander Wree**

USA

**Han-Ping Wu**

Taiwan

**Deng-Chyang Wu**

Taiwan

**Ming-Shiang Wu**

Taiwan

**Gabriele Wurm Johansson**

Sweden

**Xinhua Xiao**

China

**Guifang Xu**

China

**Shintaro Yagi**

Japan

**Kazuhito Yajima**

Japan

**Javed Yakoob**

Pakistan

**Sohsuke Yamada**

Japan

**Yutaka Yamaji**

Japan

**Hiroki Yamaue**

Japan

**Kun Yan**

China

**Hwai-I Yang**

Taiwan

**Wancai Yang**

China

**Kazuhiro Yasuda**

Japan

**Burcu Yavuz**

Turkey

**Raymond Yeung**

USA

**Jung-Hwan Yoon South**

Korea

**Shuhei Yoshida**

Japan

**Zili You**

China

**Muhammad Zubair Yousaf**

Pakistan

**Dominic Yu**

UK

**Onchee Yu**

USA

**Haiyan Zhai**

USA

**Xingqi Zhang**

China

**Lin Zhang**

China

**Qingchuan Zhao**

China

**Alexandra Zhernakova**

Netherlands

**Yan Zhong**

China

**Henning Wolfgang Zimmermann**

Germany

**Alexander Zipprich**

Germany

**Duowu Zou**

China

**Zhixiang Zuo**

USA

